# Oxidative Stress and Diabetic Retinopathy

**DOI:** 10.1155/2007/43603

**Published:** 2007-04-12

**Authors:** Renu A. Kowluru, Pooi-See Chan

**Affiliations:** Kresge Eye Institute, Wayne State University, Detroit, MI 48201, USA

## Abstract

Oxygen metabolism is essential for sustaining aerobic life, and normal cellular homeostasis works on a fine balance between the formation and elimination of reactive oxygen species (ROS). Oxidative stress, a cytopathic consequence of excessive production of ROS and the suppression of ROS removal by antioxidant defense system, is implicated in the development of many diseases, including Alzheimer's disease, and diabetes and its complications. Retinopathy, a debilitating microvascular complication of diabetes, is the leading cause of acquired blindness in developed countries. Many diabetes-induced metabolic abnormalities are implicated in its development, and appear to be influenced by elevated oxidative stress; however the exact mechanism of its development remains elusive. Increased superoxide concentration is considered as a causal link between elevated glucose and the other metabolic abnormalities important in the pathogenesis of diabetic complications. Animal studies have shown that antioxidants have beneficial effects on the development of retinopathy, but the results from very limited clinical trials are somewhat ambiguous. Although antioxidants are being used for other chronic diseases, controlled clinical trials are warranted to investigate potential beneficial effects of antioxidants in the development of retinopathy in diabetic patients.

## 1. INTRODUCTION

Diabetes, a life long progressive disease, is the result of body's inability to produce insulin or use insulin to its full potential, and is characterized by high circulating glucose. This disease has reached epidemic proportion and has become one of the most challenging health problems of the 21st century. It affects more than 230 million people worldwide, and this number is expected to reach 350 million by 2025. It is the fourth leading cause of death by disease globally; every 10 seconds a person dies from diabetes-related causes. In the United States an estimated 20.8 million people have diabetes, 14.6 million of those have been diagnosed, but 6.2 million have not yet been diagnosed. Unfortunately, the disease does not go away, but it can be controlled. A study conducted by the Centers for Disease Control suggests that diabetes care has improved over the past 10 years; but there remains a great need to focus on additional improvements because about 850 000 new cases of diabetes are diagnosed each year in United States alone.

Diabetes is a chronic disease and sustained hyperglyce-mia attacks both microvessels and macrovessels throughout the body. It is the leading cause of blindness and visual impairment, noninjury amputation, and end-stage kidney disease in adults in developed countries. It can threaten vision; patients with diabetes develop cataracts at an earlier age, and are nearly twice as likely to get glaucoma compared to nondiabetics [1]. It is the primary cause of wound healing impairments, and people with diabetes are two to four times more likely to develop cardiovascular disease than people without diabetes.

## 2. DEVELOPMENT OF DIABETIC RETINOPATHY

Diabetic retinopathy, a disease of the retina, is the leading cause of acquired blindness in working adults. The microvasculature of the retina is damaged, the blood vessels swell and leak fluid, and if not prevented, new vessels start to grow, and ultimately lead to the detachment of the retina [2, 3]. It is a duration-dependent disease that develops in stages; the incidence of retinopathy is rarely detected in the first few years of diabetes, but the incidence increases to 50% by 10 years, and to 90% by 25 years of diabetes. The prevalence of diabetic retinopathy is increasing due to prolonged survival of diabetic patients. The National Eye Institute data is very alarming; it suggests that about half of the people with diabetes in the United States have at least some form of retinopathy, and about 700 000 have some serious retinal disease. Diabetic retinopathy is affecting approximately 65 000 people in the United States alone causing 12 000 to 24 000 new cases of blindness each year.

**Figure 1:**
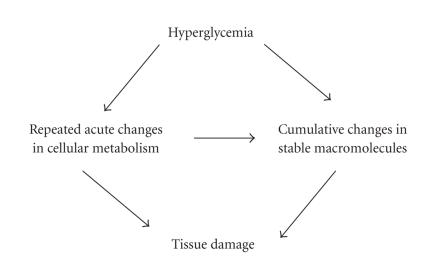
Glucose damages the retina via repeated acute and/or cumulative changes. Continued high circulating glucose in diabetes can damage retina via many acute and cumulative long-term changes that can cause tissue injury. Some acute insult, when repeated multiple times in this life-long disease, can result in cumulative changes in stable macromolecules.

Continued high circulating glucose in this life-long disease can damage retina via many acute (and repeated) and also cumulative long-term changes (Figure 1). The capillaries of retina are lined with endothelial cells that are responsible for maintaining the blood retinal barrier, and are supported with an equal number of pericytes that help provide tone to the vessels. However, in diabetes, the ratio of endothelial cells to pericytes is altered to 4 : 1 [4]. The blood vessels of retina have tight junctions that protect them from leaking, but sustained high glucose damages the tight junctions and the vessels become leaky allowing fluid or blood to seep into the retina, thus resulting in the swelling of the retina [5]. Due to progressive dysfunction, the capillaries die prematurely leading to ischemia that can be followed by neovascularization, and ultimately retinal detachment and blindness [6, 7].

In the development of diabetic retinopathy, the basement membrane thickens, the blood flow is altered, and pericytes and endothelial cells undergo accelerated apoptosis resulting in pericyte ghosts and acellular capillaries [8–12]. The leukocytes become less deformable, and retinal leukostasis is increased affecting endothelial function [13]. Although the biochemical abnormalities in the retina that are postulated to be involved in the pathogenesis of retinopathy can be seen within 2 months after induction of diabetes in rats, capillary cell apoptosis and activation of caspase-3 are observed after 6–8 months of diabetes [10, 11, 14–20]. Histopathology of diabetic retinopathy takes over decades in humans and about a year in rats to develop, and the small number of apoptotic capillary cells in diabetic retina in diabetes [10–12, 20] could have major impact on the formation of acellular capillaries and pericyte ghosts. However, these abnormalities do not present any clinical signs; the earliest clinical signs are the appearance of microaneurysms. As the disease progresses, the endothelial cells try to repair the damaged vessel by multiplying on the inner side of the vessel wall blocking the capillaries. This ultimately results in ischemia and new vessel growth. New capillaries start to grow from the surface of the retinal veins towards the center of the eye with no support, and ultimately resulting in the detachment of the retina. This suggests that the clinically silent initial phase of diabetic retinopathy consists of irreversible cellular events with late structural consequences.

In spite of extensive research, diabetic retinopathy has remained difficult to prevent and treat. Retinal photocoagulation, the procedure introduced over half a century ago, remains the best treatment for patients with diabetic retinopathy to help prevent loss of vision, but it is often not effective in restoring lost visual acuity. Photocoagulation is destructive, and can result in major adverse side effects, including loss of peripheral vision and color vision and decrease in night vision [21]. Patients with vitreous hemorrhages can undergo vitrectomy, but vitrectomy being a major surgery carries its risks. Thus, the maintenance of good glycemic control remains as one of the most effective options to prevent or delay the worsening of diabetic retinopathy. Good glycemic control can help lower the risk for developing retinopathy by 76%, and lower the risk for progression by 54% in type 1 diabetic patients. Reduction of glycated hemoglobin by only 1 unit (8% to 7%) can reduce the risk of retinopathy (and other diabetic complications) by over 30%. However, good glycemic control, for most of the patients, is difficult to achieve and to maintain for a long duration. This requires modification of behavior, dedication by the patient and the loved ones, increased risk of hypoglycemic seizure and possible weight gain [22], thus leaving the patient striving for the best possible, sensible glycemic control. An understanding of the mechanism of diabetic retinopathy is important for elucidating its pathogenesis to identify potential future therapies for treating this sight threatening disease.

### 2.1. Oxidative stress in diabetes

Under normal physiological conditions, approximately 0.1%–5% of oxygen that enters the electron transport chain is reduced to superoxide; a reactive oxygen species (ROS) and the rest are used in metabolic processes. ROS can also be generated from other sources other than the mitochondrial electron transport chain including, cytochrome P450, the NAD(P)H oxidase(s), and nitric oxide synthases [23]. ROS are produced continuously in all cells to support normal cellular functions. However, excess production of ROS originating from endogenous or exogenous sources, or inefficient removal of ROS, could result in pathological conditions. ROS produced during normal oxidative metabolism are eliminated by an efficient scavenging system, but an imbalance between production and scavenging of ROS can result in excessive levels of either molecular oxygen or ROS, thus resulting in increased “oxidative stress.” Hence, oxidative stress is the cytopathic consequence of the generation of excess ROS beyond the capacity of a cell to defend against them, and represents an imbalance between excess formation and/or impaired removal of ROS. Consequences of chronic oxidative stress include damage to biological macromolecules such as DNA, lipids, proteins, and carbohydrates, disruption in cellular homeostasis, and generation of other ROS creating further damage resulting in many disease processes of clinical interest [24].

Diabetes results in increased oxidative stress, and elevated oxidative stress plays an important role in the pathogenesis of diabetic complications [25]. Increased oxidative stress in diabetes is postulated to promote the development of neuropathy [26], nephropathy [27, 28], myocardial injury [29], and retinopathy [30]. The possible sources of oxidative stress in diabetes might include autooxidation of glucose, shifts in redox balances, decreased tissue concentrations of low molecular weight antioxidants such as reduced glutathione (GSH) and vitamin E, and impaired activities of antioxidant defense enzymes such as superoxide dismutase (SOD) and catalase [16, 31–33]. ROS generated by high glucose are considered as a causal link between elevated glucose and the other metabolic abnormalities important in the development of diabetic complications [34]. However, the exact mechanism by which oxidative stress could contribute to the development of diabetic complications still remains to be clarified.

### 2.2. Oxidative stress and diabetic retinopathy

The retina has high content of polyunsaturated fatty acids and has the highest oxygen uptake and glucose oxidation relative to any other tissue. This phenomenon renders retina more susceptible to oxidative stress [35]. It has been suggested that the correlation between hyperglycemia, changes in the redox homeostasis, and oxidative stress are the key events in the pathogenesis of diabetic retinopathy. Animal studies have demonstrated that oxidative stress contributes not only to the development of diabetic retinopathy but also to the resistance of retinopathy to reverse after good glycemic control is reinstituted—the metabolic memory phenomenon [30]. Resistance of diabetic retinopathy to reverse is probably attributed to accumulation of damaged molecules and ROS that are not easily removed even after good glycemic control is reestablished.

Superoxide levels are elevated in the retina of diabetic rats and in retinal cells incubated in high glucose media [36–38], and hydrogen peroxide content is increased in the retina of diabetic rats [39]. Membrane lipid peroxidation and oxidative damage to DNA (indicated by 8-hydroxy-2′-deoxyguanosine, 8-OHdG), the consequences of ROS-induced injury, are elevated in the retina in diabetes [16, 17, 36, 40].

Since oxidative stress represents an imbalance between excess formation and/or impaired removal of ROS, the antioxidant defense system of the cell is a crucial part of the overall oxidative stress experienced by a cell. In diabetes, the activities of antioxidant defense enzymes responsible for scavenging free radicals and maintaining redox homeostasis such as SOD, glutathione reductase, glutathione peroxidase, and catalase are diminished in the retina [16, 33]. Further, the cell is equipped with intracellular antioxidant, GSH; GSH is probably the most important defense the cell is equipped with. It can act as an ROS scavenger and modulate intracellular redox state [41]. The levels of this intracellular antioxidant are decreased in the retina in diabetes [42], and the enzymes responsible for its metabolism are compromised [43, 44]. Apart from the antioxidant defense enzymes, nonenzymic antioxidants such as vitamin C, vitamin E, and *β*-carotene that exist biologically for the regulation of redox homeostasis are also depressed during hyperglycemia-induced oxidative stress [45].

### 2.3. Oxidative stress and dysmetabolism in diabetes

**Figure 2:**
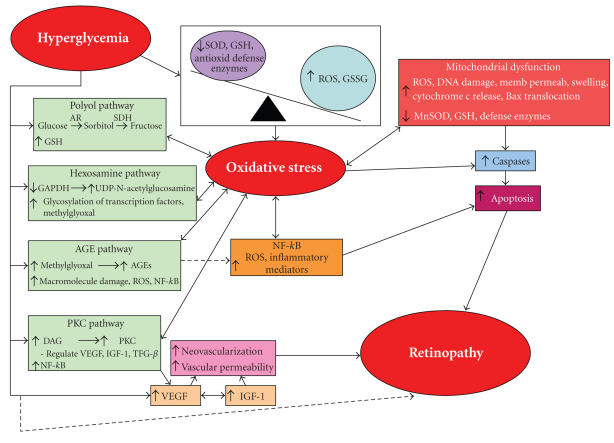
Oxidative stress-mediated dysmetabolisms in diabetic retinopathy. Oxidative stress is a cytopathic consequence of excessive production of reactive oxygen species (ROS) and the suppression of ROS removal by antioxidant defense system. Hyperglycemia-induced oxidative stress is considered a causal link between elevated glucose and other metabolic abnormalities important in the development of diabetic complications. Several diabetes-induced abnormalities in the retina that are postulated in the development of retinopathy are influenced by oxidative stress, and are considered to be interrelated.

Oxidative stress, besides creating a vicious cycle of damage to macromolecules by amplifying the production of more ROS, also activates other metabolic pathways that are detrimental to the development of diabetic retinopathy (Figure 2). These include the polyol pathway [46], the advanced glycation end product (AGE) pathway [47], protein kinase C (PKC) pathway [48, 49], the hexosamine biosynthesis pathway [50], alteration in the expressions of vascular endothelial growth factor (VEGF) [51] and insulin-like growth factor-1 (IGF-1) [52], and elevation in mitochondrial overproduction of superoxide and mitochondrial dysfunctions [53]. Several of these pathways may also mediate oxidative stress creating an interrelated connection with oxidative stress as well as with other pathways that amplify tissue damage even further. However, it has been difficult to pinpoint which pathway/s is/are critical to the development of diabetic retinopathy. More likely, no one metabolic dysfunction is the sole contributor and possibly all pathways interact to create the histopathology changes seen in diabetic retinopathy.

One of the hyperglycemia-induced metabolic perturbations in diabetic retinopathy is the polyol pathway. The pol-yol pathway involves the conversion of glucose into sorbitol, and the reaction is catalyzed by aldose reductase. Sorbitol is then oxidized to form fructose by sorbitol dehydrogenase. Increased polyol pathway in diabetes could enhance oxidative stress because aldose reductase requires NADPH, and increased polyol pathway activity is postulated to deplete NADPH by competing with glutathione reductase for NADPH. This could reduce the availability of NADPH for regenerating the intracellular antioxidant, GSH [54].

Another pathway of cellular metabolism that mediates the toxic effects of glucose is the production of AGEs. The AGEs are produced from strong glycating dicarbonyl compounds such as methylglyoxal and glyoxal [55]. Chronic hyperglycemia favors glycation reactions and nonenzymatic glycation that can lead to the alterations in function, activity, and degradation of both intracellular and extracellular proteins via chemical rearrangement and cross-linking. The AGEs formed on amino groups of proteins, lipids, and DNA can cause intramolecular and intermolecular cross-links. In diabetes, the accumulation of AGE and its receptor, RAGE, is increased in the retinal microvasculature [56]. In the late stages of retinopathy, AGEs are irreversibly formed and they accumulate within retinal capillary cells. It is postulated that more ROS are generated via the AGE pathway leading to the activation of nuclear transcriptional factor, NF-*k*B, and causing further damage to the cells [57]. The AGEs increase nitrative stress in the retinal vascular cells and initiate a sequence of events leading to retinal capillary cell apoptosis via activation of NF-*k*B and caspase-3 [58]. Nitration of proteins can inactivate mitochondrial and cytosolic proteins; disrupt protein assembly and functions, and increase apoptosis, ultimately leading to pathological consequences and damage of cellular constituents [59].

The activation of PKC is also considered as a major pathway implicated in the pathogenesis of diabetic retinopathy [49, 60, 61]. High glucose levels increase the release of ROS and the synthesis of diacylglycerol (DAG) increasing the activity of PKC [48, 62]. Activated PKC can bring about a variety of changes characteristic of diabetic retinopathy that include increasing vessel permeability, blood flow, alteration of hormone and growth factor receptor recycling, stimulation of neovascularization, endothelial proliferation and apoptosis, and regulating the action of several factors such as VEGF, IGF-1, and transforming growth factor *β* [63–65]. Inhibition of PKC activation by PKC*β* specific inhibitor (LY53331) is shown to prevent diabetes-induced oxidative stress [17, 66]. Further, recent studies by Dr. Kings's group have shown that lack of PKC*β* isoform in mice protects them from diabetes-induced oxidative stress [67]. These data suggest that oxidative stress and PKC are indeed interrelated, and support the role of PKC in ROS-mediated diabetic complications.

The hexosamine biosynthesis pathway is yet another pathway that may mediate some of the toxic effects of high glucose and superoxide concentrations in the cell [50]. The inhibition of glyceraldehyde 3 phosphate dehydrogenase (GAPDH), a multifunctional protein with diverse cytoplasmic membrane and nuclear activities, by ROS causes the diversion of all glycolytic metabolites to the hexosamine pathway producing UDP-N-acetylglucosamine which is a substrate used for the post-translational modification of intracellular factors including transcription factors [68]. Inhibition of GAPDH can result in increased levels of glycolytic metabolite glyceraldehyde 3 phosphate that can activate the AGE pathway by activating intracellular AGE precursor methylglyoxal [47, 69]. In addition, GAPDH can also be modified by direct glycation and by nitration [70, 71], and overexpression of manganese SOD (MnSOD) decreases the activation of GAPDH. Our recent results have shown that GAPDH activity is decreased in the retina obtained from diabetic rats compared to the age-matched normal control rats (Kowluru et al., unpublished observations), suggesting that GAPDH-related mechanism could be playing an important role in the pathogenesis of diabetic retinopathy.

VEGF, an angiogenesis inducer, plays a pivotal role in diabetic retinopathy and is implicated as the mediator and initiator of nonproliferative and proliferative diabetic reti-nopathies, respectively, [72, 73]. Oxidative stress mediates the hyperglycemia-induced pathological effects of VEGF on microvascular complications of diabetes [51]. Retinal expression of VEGF is elevated by ROS [74], and VEGF can also interact with other metabolic pathways important to the development of retinopathy such as PKC and the polyol pathway [75, 76].

IGF-1 can have direct mitogenic effects on endothelial cells including increased proliferation, chemotaxis, and angiogenesis, and it can stimulate glucose transport into retinal microvascular endothelial cells via activation of PKC; and can modulate the expression and activity of VEGF [52]. Similar to VEGF, the activation of IGF-1 also increases DAG levels and PKC activation [77]. Although the exact role of IGF-1 in the pathogenesis of diabetic retinopathy remains to be elucidated, it is possible that IGF-1 can be modulated by oxidative stress via PKC pathway.

There is increasing evidence to indicate ROS as mediators of pathological signal transduction pathways. ROS can serve as important downstream effectors for both Ras and Rac proteins [78]. Oxidative stress-induced Ras activation is reported to participate in the development of retinopathy in diabetes [18]. Oxidative stress can also activate a redox sensitive NF-*k*B, and NF-*k*B is also a key regulator of antioxidant enzymes [19]. Thus, activation of NF-*k*B is another plausible avenue via which oxidative stress can modulate the development of retinopathy in diabetes.

### 2.4. Oxidative stress and mitochondrial dysfunctions

During hyperglycemia glucose oxidation is increased producing an elevation in voltage gradient across the mitochondrial membrane. When a critical threshold in voltage gradient is reached, electron transfer inside complex III of the electron transport chain is blocked. The electrons accumulate at coenzyme Q that then donates them to molecular oxygen creating a lot of superoxide [79]. Mitochondria are the principal endogenous source of superoxide. Mitochondrial superoxide production initiates a cascade of damaging events via the production of more superoxide, hydrogen peroxide, hydroxyl radicals, and peroxynitrite which injure macromolecules either at or near the site of their formation [80]. Chronic overproduction of ROS in the retina results in aberrant mitochondrial functions in diabetes [53]. Hyperglycemia-induced overproduction of superoxide by the mitochondrial electron transport chain is considered to activate the major pathways of hyperglycemic damage by inhibiting GAPDH activity, and glucose-induced increase in superoxide induces mutations in mitochondrial DNA resulting in defective subunits of the electron transport complexes eventually causing increased superoxide production at physiological concentrations of glucose [34, 69]. The activity of complex III is reduced in the retinal mitochondria of diabetic mice and diabetic rats, and the levels of nitrotyrosine are elevated in the retinal mitochondria of diabetic mice compared to nondiabetic mice [81, unpublished observations].

One of the ROS-induced dysfunctions in mitochondria is the repression of antioxidant defense capabilities that could lead to enhanced sensitivity of retinal cells to oxidative stress because they cannot scavenge ROS effectively. The isoform of SOD in the mitochondria, MnSOD, together with GSH, is suppressed in the diabetic and high glucose-cultured retinal mitochondria [81–83]. Mitochondrial dysfunction also includes damage to mitochondrial DNA [84], and mitochondrial DNA damage is increased in the retina in diabetes [81]. Damage to the mitochondrial lipid membrane by ROS increases the permeability of the organelle, and the modulation of the permeability transition of mitochondrial membrane represents another dysfunction caused by ROS. Increased swelling of the mitochondria is observed in the retina of diabetic mice [81]. The inner mitochondrial membrane space contains several soluble proteins including cytochrome c; the release of cytochrome c from mitochondria to the cytoplasm and Bax translocation from the cytosol to mitochondria that could drive cell apoptosis are increased in the retina and its capillary cells in diabetes [36].

Thus, it is evident that oxidative stress can modulate mitochondria function resulting in increased apoptosis of retinal capillary cells; however, additional studies to determine the role of oxidative stress-induced mitochondrial dysfunctions in diabetic retinopathy are warranted.

### 2.5. Oxidative stress and apoptosis

It is widely known that apoptosis of retinal cells is a consummated phenomenon in diabetic retinopathy. Retinal capillary cells undergo accelerated apoptosis that precedes the detection of any histopathology changes characteristic of this diabetes complication [10, 11]. Exposure of the pericytes and endothelial cells to high glucose or diabetic animals showed an increase in oxidative stress, caspase-3 activity, and other transcription factors leading to capillary cell death [16, 19, 20, 85]. Terminal transferase dUTP nick end labeling (TUNEL) positive cells are observed in rat and mice retinal microvasculature at 6 to 8 months of diabetes [10–12, 86]. The histological evidence of apoptosis is also supported by some of the biochemical observations that demonstrated an increase in the expression of Bax in the diabetic retina [87]. These findings substantiate that apoptosis of retinal capillary cells is mediated through sequential events.

Retinal Muller cells, ganglion cells, astrocytes, and photoreceptors are also affected early on in the course of the development of diabetic retinopathy [88–90], and their role in the pathogenesis of diabetic retinopathy is being investigated by several other laboratories. Although the exact signaling steps to retinal capillary cells apoptosis in diabetic retinopathy remain unclear, the results have pointed to the involvement of oxidative stress-activated caspases and NF-*k*B in retinal cell death [19, 20], and inhibition of superoxide accumulation in diabetes prevents apoptosis of retinal capillary cells [36, 82, 83]. The mechanism by which oxidative stress can increase apoptosis appears to be complex, but could involve increases in membrane lipid peroxidation and oxidative injury to the macromolecules essential for cellular functions, and alterations in signal transduction and gene expression [91, 92]. ROS can indirectly induce apoptosis by changing cellular redox potentials, depleting GSH and reducing ATP levels [93]. In retinal pericytes obtained from diabetic patients, the altered gene profile of scavenging enzymes correlates with the overexpression of the cell death protease gene, suggesting an important role of oxidative stress in pericyte dropout seen in diabetic retinopathy [94]. The release of ROS increases mitochondrial pore permeability that in turn triggers the release of cytochrome c and other proapoptotic factors from retinal mitochondria initiating apoptosis via activation of caspases [95, 96], and increased cytochrome c is observed in the retina and its capillary cells in diabetes [36, 53, 83].

Caspases, a group of cysteine proteases that are essential for mediating apoptosis in cells [97], are known to be very sensitive toward oxidative and nitrative stress [98]. ROS-induced mitochondrial dysfunction pertaining to the release of cytochrome c can result in activation of caspase-9 which initiates a cascade of events that activates caspase-3 responsible for fragmenting DNA [96, 99]. Caspase-3 is activated in the retina in diabetes, and the therapy that inhibits the development of retinopathy in diabetic rats also inhibits retinal caspase-3 activation [20], suggesting that increased oxidative stress can modulate retinal cell apoptosis in diabetes via caspase-3 pathway.

Another apoptosis execution mediator is the redox sensitive-NF-*k*B. Although the effects of NF-*k*B activation can be either anti- or proapoptotic depending on the cell type and disease state, diabetes-induced activation of NF-*k*B in the retina and its capillary cells is considered to be proapoptotic [19, 87]. The activation of NF-*k*B is considered a key signaling pathway by which high glucose induces apoptosis in endothelial cells [100]. Diabetes-induced NF-*k*B activation is reported to trigger a proapoptotic program in retinal pericytes [101]. We have shown that NF-*k*B is activated in endothelial cells and pericytes incubated in high glucose medium, and in retina in diabetes before either cell death or histopathology can be seen, and this continues during the time when the histopathology is developing, suggesting that the activation of NF-*k*B is an early event in the development of diabetic retinopathy [19]. Activation of NF-*k*B modulates the expression of several proinflammatory factors, including tumor necrosis factor and inducible nitric oxide synthase, and this, in turn, can result in increased free radical production [102]. Reaction between superoxide and nitric oxide (NO) forms peroxynitrite, which can increase DNA damage, induce formation of 8-OHdG, and deplete intracellular GSH levels. Peroxynitrite is a powerful oxidant that can react with a wide range of targets to cause oxidation of membrane phospholipids, protein and nonprotein thiols, results in single-strand DNA breaks, and nitrates tyrosine residues [103]. It can injure mitochondria leading to increase in mitochondrial pore opening and consequently apoptosis [104, 105]. Peroxynitrite levels are elevated in retina early in diabetes and remain elevated at 14 months of diabetes in rats [19, 106], and increased nitrotyrosine can be localized in the retinal vasculature of diabetic rats [107].

### 2.6. Oxidative stress and inflammation

Diabetic retinopathy shares similarities with chronic inflammatory disease [108], and inflammation may play a central role in the development and progression of diabetic retinopathy. ROS is considered as a strong stimulus for the release of cytokines [109], and increased superoxide can promote inflammation through various pathways; they can damage endothelial cells, increase microvascular permeability and release cytokines, and help in the recruitment of neutrophils at the site of inflammation [110]. Thus, the role of oxidative stress in the inflammation-mediated development of diabetic retinopathy needs further investigation.

The activation of NF-*k*B by ROS could increase proinflammatory mediators such as the cytokines, NO, and pros-taglandins [111]. The levels of cytokines including interleukin (IL)-1*β*, IL-6, and IL-8 are increased in the vitreous fluid of patients with proliferative diabetic retinopathy [112] and in the retina of diabetic rats and mice [113, 114]. The levels of IL-1*β* are increased substantially also in retinal capillary cells incubated in high glucose media [114]. Stimulation of IL-1 can lead to the release of more ROS and NF-*k*B activation, and this could create a continuous feedback loop [109, 115]. We have shown that IL-1*β* administration into the vitreous of normal rats increases oxidative stress in the retina and this increase is similar to that observed in diabetes [114, 116]. The apoptosis of retinal capillary cells also increases with IL-1*β*, and this is believed to be mediated by the activation of NF-*k*B and caspase-3 [106, 114, 116].

Cyclooxygenase-2 (COX-2) that catalyzes the formation of prostaglandin E_2_ (PGE_2_) is induced by IL-1, and COX-2 and PGE_2_ are reported to contribute to the development of diabetic retinopathy by modulating VEGF-mediated vascular permeability and angiogenesis [117]. Thus, oxidative stress may directly or indirectly induce the release of inflammatory mediators and the inflammation process implicated in the pathogenesis of diabetic retinopathy.

## 3. TREATMENTS FOR DIABETIC RETINOPATHY

Since there remains a strong understanding that oxidative stress may be the instigator of all other dysmetabolisms implicated in the pathogenesis of diabetic retinopathy, the use of appropriate antioxidants may have potential on the metabolic and functional abnormalities in diabetic retinopathy. Antioxidants may act at different levels; they may inhibit the formation of ROS or scavenge free radicals, or increase the antioxidants defense enzyme capabilities.

Lipoic acid is an antioxidant capable of thiol-disulfide exchange. It is able to scavenge ROS and reduce metabolites such as glutathione to maintain a healthy cellular redox state [118]. It distributes to the mitochondria and serves as a critical cofactor for the mitochondrial enzyme complexes, and is regenerated via glycolytic flux. Lipoic acid attenuates the apoptosis of rat retinal capillary cells and decreases the levels of 8-OHdG and nitrotyrosine [12]. Lipoic acid supplementation completely prevents diabetes-induced increase in nitrotyrosine and activation of NF-*k*B while decreasing the levels of VEGF and oxidatively modified proteins in the rat retina [12, 119]. This antioxidant also inhibits diabetes-induced decreases in retinal mitochondrial and cytosolic ratios of NAD^+^ to NADH [120]. We have shown that long-term administration of lipoic acid prevents the development of diabetic retinopathy in rats, the number of apoptotic capillary cells and acellular capillaries is decreased in the retina of diabetic rats [12].

Benfotiamine, a lipid soluble thiamine (vitamin B1) de-rivative that inhibits MnSOD, has been shown to inhibit increases in acellular capillaries in the retina of diabetic rats via blocking the major pathways involved in hyperglycemia-induced retinal dysmetabolism, including AGEs, PKC, and hexosamine pathways [121].

Green tea, rich in polyphenols with great antioxidant potency, inhibits lipid peroxidation, and scavenges hydroxyl and superoxide radicals [122]. Green tea supplementation in diabetic rats is reported to improve the levels of SOD and GSH, reduce the serum glucose levels, and improve retinopathy as evident by reductions in acellular capillaries and pericyte ghosts [123]. This provides encouraging rationale for its possible therapeutic use to inhibit retinopathy in diabetic patients.

Trolox is a water soluble analog of vitamin E with potent antioxidant properties. Trolox is shown to partially prevent the loss of pericytes in diabetic rats via reducing membrane lipid peroxidation [124]. However, no additional followup studies have been reported by either the same group or other investigators.

Nicanartine, an antioxidant with cholesterol lowering properties, can partially inhibit pericyte loss in diabetic rats. However, in the same animals it fails to provide any benefit in normalizing diabetes-induced increase in retinal acellular capillaries [125].

Zinc, a trace element with antioxidant properties, is shown to prevent diabetes-induced glutathione loss in the retina [126]. Further, another trace element, selenium, is reported to down-regulate VEGF production in the retina in diabetes [127].

Dietary supplementation with multiantioxidants comprising of vitamins C and E in diabetic rats prevents inhibition in retinal glutathione reductase, glutathione peroxidase, and SOD activities [44]. Superoxide production in the retina is repressed by the same combination of vitamins in diabetic rats [123]. Partial reductions in the development of retinal acellular capillaries and pericyte ghosts are seen in diabetic rats given the combination of vitamins C and E [16]. In another study, the same combination of antioxidants is shown to decrease pericyte dropout significantly in the retina of diabetic rats [123]. The benefits pertaining to retinal cells survival are more profound in diabetic rats consuming multiantioxidants containing more components including ascorbic acid, *α*-tocopherol acetate, Trolox, N-acetyl cysteine, *β*-carotene, and selenium. Besides decreasing microvascular lesions, the multiantioxidants abrogate the diabetes-induced increases in retinal PKC and NO [16]. The same components of multiantioxidants other than decreasing retinal PKC activity also reduce lipid peroxide, and prevent the decrease in SOD, glutathione reductase, and catalase activities [128]. Thus, by increasing the diversity of antioxidants, retinopathy is better prevented in the animal models of diabetic retinopathy.

Our recent studies using genetic manipulation techniques have shown that overexpression of mitochondrial SOD in mice can prevent diabetes-induced decrease in retinal oxidative stress, and protect the mitochondria from dysfunction [83], this raises the possibility that MnSOD mimics could provide an attractive pharmacological approach to inhibit the development of diabetic retinopathy.

Thus, there is accumulating evidence from animal studies that oxidative stress is associated with the development of retinopathy in diabetes, and antioxidants have beneficial effects on the development of retinopathy. However, the results from clinical trials are ambiguous. Calcium dobesilate (2,5-dihydroxybenzenesulfonate), a compound with potent antioxidant capacity against hydroxyl radical, and a registered compound for the treatment of diabetic retinopathy, is shown to reduce the progression of this sight-threatening complication of diabetes [129]. Pycnogenol, a compound with both free radical scavenging and antiinflammatory properties, is also reported to have beneficial effects on the progression of retinopathy in diabetic patients [130]. Vitamin E treatment in clinical trials has been shown to inhibit diabetes-induced retinal hemodynamics [131].

In contrast, others have found no significant associations between serum levels of major dietary antioxidants and retinopathy in type 2 diabetic patients [132, 133] and a single 24-hour diet recall study has provided no beneficial effects of supplementation with vitamins C, E, and *β*-carotene [134]. The differences for such discrepancies are not clear, but it is possible that the initiation of antioxidants could be subsequent to the development of background retinopathy, in contrast to the animal studies where antioxidants have been administered soon after establishment of diabetes, and additional trials need to be initiated. Or, this could be that the antioxidant concentrations in the retina were not sufficient to produce beneficial effects. These over the counter antioxidants appear to be promising in inhibiting the development of diabetic retinopathy in animal models. But further clinical studies are needed to determine the appropriate regimen, and also whether these therapies could have long-term effects that may help diabetic patients to slow the progression of this sight-threatening complication of diabetes. The clinicians need to caution the patients so that the patients are made aware of possible shortcomings before initiating such therapies.

We need to recognize that since diabetes-induced metabolic abnormalities are interrelated in retina [17], inhibiting a single detrimental pathway of retinal metabolism in diabetes might have multiple beneficial effects on retinopathy. But, if the treatment does not completely inhibit the targeted metabolic abnormality, this could result in partial inhibition of other interrelated abnormalities. Thus, we might not have one single drug that could effectively treat this complication of diabetes, and it may be prudent to use a group of drugs with divergent mode(s) of action to combat this multifactorial complication, and antioxidants could be an integral part of that regimen.

## DEFINITIONS

AGE: Advanced glycation end product
AR: Aldose reductase
COX-2: Cyclooxygenase-2
DAG: Diacylglycerol
GAPDH: Glyceraldehyde 3 phosphate dehydrogenase
GSSG: Oxidized glutathione
GSH: Reduced glutathione
IGF-1: Insulin-like growth factor-1
IL: Interleukin
MnSOD: Manganese superoxide dismutase
NF-*k*B: Nuclear transcription factor
NO: Nitric oxide
8-OHdG: 8-hydroxy-2'-deoxyguanosine
PGE_2_: Prostaglandin E_2_
PKC: Protein kinase C
ROS: Reactive oxygen species
SDH: Sorbitol dehydrogenase
SOD: Superoxide dismutase
TGF-*β*: Transforming growth factor-*β*
VEGF: Vascular endothelial growth factor
TUNEL: Terminal transferase dUTP nick end labeling

## References

[1] Sharma S, Oliver-Fernandez A, Liu W, Buchholz P, Walt J (2005). The impact of diabetic retinopathy on health-related quality of life. *Current Opinion in Ophthalmology*.

[2] Frank RN (2004). Diabetic retinopathy. *New England Journal of Medicine*.

[3] Aylward GW (2005). Progressive changes in diabetics and their management. *Eye*.

[4] Robison WG, Kador PF, Kinoshita JH (1985). Early retinal microangiopathy: prevention with aldose reductase inhibitors. *Diabetic Medicine*.

[5] Harhaj NS, Antonetti DA (2004). Regulation of tight junctions and loss of barrier function in pathophysiology. *International Journal of Biochemistry and Cell Biology*.

[6] Aiello LP, Gardner TW, King GL (1998). Diabetic retinopathy. *Diabetes Care*.

[7] Frank RN (1991). On the pathogenesis of diabetic retinopathy: a 1990 update. *Ophthalmology*.

[8] Engerman RL, Davis MD, Bloodworth JMB, Rodriguez R, Vallance-Owen J (1971). Retinopathy in experimental diabetes: its relevance to diabetic retinopathy in man. *Diabetes, Proceedings of the 7th Congress of the International Diabetes Federation*.

[9] Kern TS, Kowluru RA, Engerman RL (1994). *Questions Raised by Studies of Experimental Diabetic Retinopathy*.

[10] Mizutani M, Kern TS, Lorenzi M (1996). Accelerated death of retinal microvascular cells in human and experimental diabetic retinopathy. *Journal of Clinical Investigation*.

[11] Kern TS, Tang J, Mizutani M (2000). Response of capillary cell death to aminoguanidine predicts the development of retinopathy: comparison of diabetes and galactosemia. *Investigative Ophthalmology & Visual Science*.

[12] Kowluru RA, Odenbach S (2004). Effect of long-term administration of *α*-lipoic acid on retinal capillary cell death and the development of retinopathy in diabetic rats. *Diabetes*.

[13] Joussen AM, Poulaki V, Le ML (2004). A central role for inflammation in the pathogenesis of diabetic retinopathy. *FASEB Journal*.

[14] Xia P, Inoguchi T, Kern TS, Engerman RL, Oates PJ, King GL (1994). Characterization of the mechanism for the chronic activation of diacylglycerol-protein kinase C pathway in diabetes and hypergalactosemia. *Diabetes*.

[15] Kowluru RA, Jirousek MR, Stramm L, Farid N, Engerman RL, Kern TS (1998). Abnormalities of retinal metabolism in diabetes or experimental galactosemia. V. Relationship between protein kinase C and APTases. *Diabetes*.

[16] Kowluru RA, Tang J, Kern TS (2001). Abnormalities of retinal metabolism in diabetes and experimental galactosemia. VII. Effect of long-term administration of antioxidants on the development of retinopathy. *Diabetes*.

[17] Kowluru RA (2001). Diabetes-induced elevations in retinal oxidative stress, protein kinase C and nitric oxide are interrelated. *Acta Diabetologica*.

[18] Kowluru RA, Kowluru A, Chakrabarti S, Khan Z (2004). Potential contributory role of H-Ras, a small G-protein, in the development of retinopathy in diabetic rats. *Diabetes*.

[19] Kowluru RA, Koppolu P, Chakrabarti S, Chen S (2003). Diabetes-induced activation of nuclear transcriptional factor in the retina, and its inhibition by antioxidants. *Free Radical Research*.

[20] Kowluru RA, Koppolu P (2002). Diabetes-induced activation of caspase-3 in retina: effect of antioxidant therapy. *Free Radical Research*.

[21] Bhavsar AR (2006). Diabetic retinopathy: the latest in current management. *Retina*.

[22] Diabetes Control and Complications Trial Research Group (1997). Hypoglycemia in the diabetes control and complications trial. *Diabetes*.

[23] Dröge W (2002). Free radicals in the physiological control of cell function. *Physiological Reviews*.

[24] Cutler RG (2005). Oxidative stress profiling—part I. Its potential importance in the optimization of human health. *Annals of the New York Academy of Sciences*.

[25] Baynes JW (1991). Role of oxidative stress in development of complications in diabetes. *Diabetes*.

[26] Feldman EL (2003). Oxidative stress and diabetic neuropathy: a new understanding of an old problem. *Journal of Clinical Investigation*.

[27] Ha H, Kim KH (1999). Pathogenesis of diabetic nephropathy: the role of oxidative stress and protein kinase C. *Diabetes Research and Clinical Practice*.

[28] Hinokio Y, Suzuki S, Hirai M, Suzuki C, Suzuki M, Toyota T (2002). Urinary excretion of 8-oxo-7, 8-dihydro-2′-deoxyguanosine as a predictor of the development of diabetic nephropathy. *Diabetologia*.

[29] Cai L, Kang YJ (2001). Oxidative stress and diabetic cardiomyopathy: a brief review. *Cardiovascular Toxicology*.

[30] Kowluru RA (2003). Effect of reinstitution of good glycemic control on retinal oxidative stress and nitrative stress in diabetic rats. *Diabetes*.

[31] Wohaieb SA, Godin DV (1987). Alterations in free radical tissue-defense mechanisms in streptozocin-induced diabetes in rat. Effects of insulin treatment. *Diabetes*.

[32] Baynes JW, Thorpe SR (1999). Role of oxidative stress in diabetic complications: a new perspective on an old paradigm. *Diabetes*.

[33] Haskins K, Bradley B, Powers K (2003). Oxidative stress in type 1 diabetes. *Annals of the New York Academy of Sciences*.

[34] Brownlee M (2001). Biochemistry and molecular cell biology of diabetic complications. *Nature*.

[35] Anderson RE, Rapp LM, Wiegand RD (1984). Lipid peroxidation and retinal degeneration. *Current Eye Research*.

[36] Kowluru RA, Abbas SN (2003). Diabetes-induced mitochondrial dysfunction in the retina. *Investigative Ophthalmology & Visual Science*.

[37] Du Y, Miller CM, Kern TS (2003). Hyperglycemia increases mitochondrial superoxide in retina and retinal cells. *Free Radical Biology and Medicine*.

[38] Cui Y, Xu X, Bi H (2006). Expression modification of uncoupling proteins and MnSOD in retinal endothelial cells and pericytes induced by high glucose: the role of reactive oxygen species in diabetic retinopathy. *Experimental Eye Research*.

[39] Ellis EA, Guberski DL, Somogyi-Mann M, Grant MB (2000). Increased H_2_O_2_, vascular endothelial growth factor and receptors in the retina of the BBZ/WOR diabetic rat. *Free Radical Biology and Medicine*.

[40] Kowluru RA, Koppolu P (2002). Termination of experimental galactosemia in rats, and progression of retinal metabolic abnormalities. *Investigative Ophthalmology & Visual Science*.

[41] Meister A (1988). Glutathione metabolism and its selective modification. *Journal of Biological Chemistry*.

[42] Kern TS, Kowluru RA, Engerman RL (1994). Abnormalities of retinal metabolism in diabetes or galactosemia: ATPases and glutathione. *Investigative Ophthalmology & Visual Science*.

[43] Kowluru RA, Kern TS, Engerman RL (1994). Abnormalities of retinal metabolism in diabetes or galactosemia II. Comparison of *γ*-glutamyl transpeptidase in retina and cerebral cortex, and effects of antioxidant therapy. *Current Eye Research*.

[44] Kowluru RA, Kern TS, Engerman RL (1996). Abnormalities of retinal metabolism in diabetes or experimental galactosemia. IV. Antioxidant defense system. *Free Radical Biology and Medicine*.

[45] Ford ES, Mokdad AH, Giles WH, Brown DW (2003). The metabolic syndrome and antioxidant concentrations: findings from the Third National Health and Nutrition Examination Survey. *Diabetes*.

[46] Engerman RL, Kern TS, Larson ME (1994). Nerve conduction and aldose reductase inhibition during 5 years of diabetes or galactosaemia in dogs. *Diabetologia*.

[47] Beisswenger PJ, Howell SK, Smith K, Szwergold BS (2003). Glyceraldehyde-3-phosphate dehydrogenase activity as an independent modifier of methylglyoxal levels in diabetes. *Biochimica et Biophysica Acta (BBA) - Molecular Basis of Disease*.

[48] Stauble B, Boscoboinik D, Tasinato A, Azzi A (1994). Modulation of activator protein-1 (AP-1) transcription factor and protein kinase C by hydrogen peroxide and D-*α*-tocopherol in vascular smooth muscle cells. *European Journal of Biochemistry*.

[49] Koya D, King GL (1998). Protein kinase C activation and the development of diabetic complications. *Diabetes*.

[50] Du X-L, Edelstein D, Rossetti L (2000). Hyperglycemia-induced mitochondrial superoxide overproduction activates the hexosamine pathway and induces plasminogen activator inhibitor-1 expression by increasing Sp1 glycosylation. *Proceedings of the National Academy of Sciences of the United States of America*.

[51] Caldwell RB, Bartoli M, Behzadian MA (2005). Vascular endothelial growth factor and diabetic retinopathy: role of oxidative stress. *Current Drug Targets*.

[52] DeBosch BJ, Baur E, Deo BK, Hiraoka M, Kumagai AK (2001). Effects of insulin-like growth factor-1 on retinal endothelial cell glucose transport and proliferation. *Journal of Neurochemistry*.

[53] Kowluru RA (2005). Diabetic retinopathy: mitochondrial dysfunction and retinal capillary cell death. *Antioxidants & Redox Signaling*.

[54] Miwa K, Nakamura J, Hamada Y (2003). The role of polyol pathway in glucose-induced apoptosis of cultured retinal pericytes. *Diabetes Research and Clinical Practice*.

[55] Glomb MA, Monnier VM (1995). Mechanism of protein modification by glyoxal and glycolaldehyde, reactive intermediates of the Maillard reaction. *Journal of Biological Chemistry*.

[56] Stitt AW (2003). The role of advanced glycation in the pathogenesis of diabetic retinopathy. *Experimental and Molecular Pathology*.

[57] Mohamed AK, Bierhaus A, Schiekofer S, Tritschler H, Ziegler R, Nawroth PP (1999). The role of oxidative stress and NF-*κ*B activation in late diabetic complications. *BioFactors*.

[58] Kowluru RA (2005). Effect of advanced glycation end products on accelerated apoptosis of retinal capillary cells under in vitro conditions. *Life Sciences*.

[59] Cowell RM, Russell JW (2004). Nitrosative injury and antioxidant therapy in the management of diabetic neuropathy. *Journal of Investigative Medicine*.

[60] Ishii H, Jirousek MR, Koya D (1996). Amelioration of vascular dysfunctions in diabetic rats by an oral PKC *β* inhibitor. *Science*.

[61] Kowluru RA, Kern TS, Engerman RL, Armstrong D (1996). Abnormalities of retinal metabolism in diabetes or experimental galactosemia. III. Effects of antioxidants. *Diabetes*.

[62] Palumbo EJ, Sweatt JD, Chen S-J, Klann E (1992). Oxidation-induced persistent activation of protein kinase C in hippocampal homogenates. *Biochemical and Biophysical Research Communications*.

[63] Oikawa T, Shimamura M, Ashino H (1992). Inhibition of angiogenesis by staurosporine, a potent protein kinase inhibitor. *Journal of Antibiotics*.

[64] Xia P, Aiello LP, Ishii H (1996). Characterization of vascular endothelial growth factor's effect on the activation of protein kinase C, its isoforms, and endothelial cell growth. *Journal of Clinical Investigation*.

[65] Koya D, Jirousek MR, Lin Y-W, Ishii H, Kuboki K, King GL (1997). Characterization of protein kinase C *β* isoform activation on the gene expression of transforming growth factor-*β*, extracellular matrix components, and prostanoids in the glomeruli of diabetic rats. *Journal of Clinical Investigation*.

[66] Wu Y, Wu G, Qi X (2006). Protein kinase C *β* inhibitor LY333531 attenuates intercellular adhesion molecule-1 and monocyte chemotactic protein-1 expression in the kidney in diabetic rats. *Journal of Pharmacological Sciences*.

[67] Ohshiro Y, Ma RC, Yasuda Y (2006). Reduction of diabetes-induced oxidative stress, fibrotic cytokine expression, and renal dysfunction in protein kinase C*β*-null mice. *Diabetes*.

[68] Du X, Matsumura T, Edelstein D (2003). Inhibition of GAPDH activity by poly(ADP-ribose) polymerase activates three major pathways of hyperglycemic damage in endothelial cells. *Journal of Clinical Investigation*.

[69] Brownlee M (2005). The pathobiology of diabetic complications: a unifying mechanism. *Diabetes*.

[70] Beisswenger PJ, Drummond KS, Nelson RG, Howell SK, Szwergold BS, Mauer M (2005). Susceptibility to diabetic nephropathy is related to dicarbonyl and oxidative stress. *Diabetes*.

[71] Ishii T, Sunami O, Nakajima H, Nishio H, Takeuchi T, Hata F (1999). Critical role of sulfenic acid formation of thiols in the inactivation of glyceraldehyde-3-phosphate dehydrogenase by nitric oxide. *Biochemical Pharmacology*.

[72] Lutty GA, McLeod DS, Merges C, Diggs A, Plouét J (1996). Localization of vascular endothelial growth factor in human retina and choroid. *Archives of Ophthalmology*.

[73] Aiello LP, Wong J-S (2000). Role of vascular endothelial growth factor in diabetic vascular complications. *Kidney International*.

[74] Lu M, Kuroki M, Amano S (1998). Advanced glycation end products increase retinal vascular endothelial growth factor expression. *Journal of Clinical Investigation*.

[75] Aiello LP, Bursell S-E, Clermont A (1997). Vascular endothelial growth factor-induced retinal permeability is mediated by protein kinase C in vivo and suppressed by an orally effective *β*-isoform-selective inhibitor. *Diabetes*.

[76] Frank RN, Amin R, Kennedy A, Hohman TC (1997). An aldose reductase inhibitor and aminoguanidine prevent vascular endothelial growth factor expression in rats with long-term galactosemia. *Archives of Ophthalmology*.

[77] Yano K, Bauchat JR, Liimatta MB, Clemmons DR, Duan C (1999). Down-regulation of protein kinase C inhibits insulin-like growth factor I-induced vascular smooth muscle cell proliferation, migration, and gene expression. *Endocrinology*.

[78] Finkel T (2006). Intracellular redox regulation by the family of small GTPases. *Antioxidants & Redox Signaling*.

[79] Young TA, Cunningham CC, Bailey SM (2002). Reactive oxygen species production by the mitochondrial respiratory chain in isolated rat hepatocytes and liver mitochondria: studies using myxothiazol. *Archives of Biochemistry and Biophysics*.

[80] Bergamini CM, Gambetti S, Dondi A, Cervellati C (2004). Oxygen, reactive oxygen species and tissue damage. *Current Pharmaceutical Design*.

[81] Kanwar M, Chan P-S, Kern TS, Kowluru RA Oxidative damage in the retinal mitochondria of diabetic mice: possible protection by superoxide dismutase.

[82] Kowluru RA, Atasi L, Ho YS (2006). Role of mitochondrial superoxide dismutase in the development of diabetic retinopathy. *Investigative Ophthalmology & Visual Science*.

[83] Kowluru RA, Kowluru V, Xiong Y, Ho Y-S (2006). Overexpression of mitochondrial superoxide dismutase in mice protects the retina from diabetes-induced oxidative stress. *Free Radical Biology and Medicine*.

[84] Maassen JA, 'T Hart LM, Van Essen E (2004). Mitochondrial diabetes: molecular mechanisms and clinical presentation. *Diabetes*.

[85] Kowluru RA, Abbas SN, Odenbach S (2004). Reversal of hyperglycemia and diabetic nephropathy: effect of reinstitution of good metabolic control on oxidative stress in the kidney of diabetic rats. *Journal of Diabetes and Its Complications*.

[86] Feit-Leichman RA, Kinouchi R, Takeda M (2005). Vascular damage in a mouse model of diabetic retinopathy: relation to neuronal and glial changes. *Investigative Ophthalmology & Visual Science*.

[87] Podesta F, Romeo G, Liu W-H (2000). Bax is increased in the retina of diabetic subjects and is associated with pericyte apoptosis in vivo and in vitro. *American Journal of Pathology*.

[88] Barber AJ, Lieth E, Khin SA, Antonetti DA, Buchanan AG, Gardner TW (1998). Neural apoptosis in the retina during experimental and human diabetes: early onset and effect of insulin. *Journal of Clinical Investigation*.

[89] Mizutani M, Gerhardinger C, Lorenzi M (1998). Muller cell changes in human diabetic retinopathy. *Diabetes*.

[90] Phipps JA, Fletcher EL, Vingrys AJ (2004). Paired-flash identification of rod and cone dysfunction in the diabetic rat. *Investigative Ophthalmology & Visual Science*.

[91] Matsura T, Kai M, Fujii Y, Ito H, Yamada K (1999). Hydrogen peroxide-induced apoptosis in HL-60 cells requires caspase-3 activation. *Free Radical Research*.

[92] Kaneto H, Kajimoto Y, Miyagawa J (1999). Beneficial effects of antioxidants in diabetes: possible protection of pancreatic *β*-cells against glucose toxicity. *Diabetes*.

[93] Hancock JT, Desikan R, Neill SJ (2001). Does the redox status of cytochrome C act as a fail-safe mechanism in the regulation of programmed cell death?. *Free Radical Biology and Medicine*.

[94] Li W, Yanoff M, Jian B, He Z (1999). Altered mRNA levels of antioxidant enzymes in pre-apoptotic pericytes from human diabetic retinas. *Cellular and Molecular Biology*.

[95] Anuradha CD, Kanno S, Hirano S (2001). Oxidative damage to mitochondria is a preliminary step to caspase-3 activation in fluoride-induced apoptosis in HL-60 cells. *Free Radical Biology and Medicine*.

[96] Phaneuf S, Leeuwenburgh C (2002). Cytochrome *c* release from mitochondria in the aging heart: a possible mechanism for apoptosis with age. *American Journal of Physiology - Regulatory Integrative and Comparative Physiology*.

[97] Alnemri ES (1997). Mammalian cell death proteases: a family of highly conserved aspartate specific cysteine proteases. *Journal of Cellular Biochemistry*.

[98] Mohr S, Zech B, Lapetina EG, Brüne B (1997). Inhibition of caspase-3 by S-Nitrosation and oxidation caused by nitric oxide. *Biochemical and Biophysical Research Communications*.

[99] Kristal BS, Koopmans SJ, Jackson CT, Ikeno Y, Park B-J, Yu BP (1997). Oxidant-mediated repression of mitochondrial transcription in diabetic rats. *Free Radical Biology and Medicine*.

[100] Du X, Stockklauser-Färber K, Rösen P (1999). Generation of reactive oxygen intermediates, activation of NF-*κ*B, and induction of apoptosis in human endothelial cells by glucose: role of nitric oxide synthase?. *Free Radical Biology and Medicine*.

[101] Romeo G, Liu W-H, Asnaghi V, Kern TS, Lorenzi M (2002). Activation of nuclear factor-*κ*B induced by diabetes and high glucose regulates a proapoptotic program in retinal pericytes. *Diabetes*.

[102] Griscavage JM, Wilk S, Ignarro LJ (1996). Inhibitors of the proteasome pathway interfere with induction of nitric oxide synthase in macrophages by blocking activation of transcription factor NF-*κ*B. *Proceedings of the National Academy of Sciences of the United States of America*.

[103] Beckman JS, Koppenol WH (1996). Nitric oxide, superoxide, and peroxynitrite: the good, the bad, and the ugly. *American Journal of Physiology - Cell Physiology*.

[104] Behar-Cohen FF, Heydolph S, Faure V, Droy-Lefaix M-T, Courtois Y, Goureau O (1996). Peroxynitrite cytotoxicity on bovine retinal pigmented epithelial cells in culture. *Biochemical and Biophysical Research Communications*.

[105] Radi R, Cassina A, Hodara R, Quijano C, Castro L (2002). Peroxynitrite reactions and formation in mitochondria. *Free Radical Biology and Medicine*.

[106] Kowluru RA, Chakrabarti S, Chen S (2004). Re-institution of good metabolic control in diabetic rats and activation of caspase-3 and nuclear transcriptional factor (NF-*k*B) in the retina. *Acta Diabetologica*.

[107] Du Y, Smith MA, Miller CM, Kern TS (2002). Diabetes-induced nitrative stress in the retina, and correction by aminoguanidine. *Journal of Neurochemistry*.

[108] Joussen AM, Murata T, Tsujikawa A, Kirchhof B, Bursell S-E, Adamis AP (2001). Leukocyte-mediated endothelial cell injury and death in the diabetic retina. *American Journal of Pathology*.

[109] Chang CK, LoCicero J (2004). Overexpressed nuclear factor *κ*B correlates with enhanced expression of interleukin-1*β* and inducible nitric oxide synthase in aged murine lungs to endotoxic stress. *Annals of Thoracic Surgery*.

[110] Quan N, He L, Lai W (2003). Endothelial activation is an intermediate step for peripheral lipopolysaccharide induced activation of paraventricular nucleus. *Brain Research Bulletin*.

[111] Schreck R, Albermann K, Baeuerle PA (1992). Nuclear factor *κ*
*β*: an oxidative stress-responsive transcription factor of eukaryotic cells (a review). *Free Radical Research Communications*.

[112] Yuuki T, Kanda T, Kimura Y (2001). Inflammatory cytokines in vitreous fluid and serum of patients with diabetic vitreoretinopathy. *Journal of Diabetes and Its Complications*.

[113] Carmo A, Cunha-Vaz JG, Carvalho AP, Lopes MC (1999). L-arginine transport in retinas from streptozotocin diabetic rats: correlation with the level of IL-1*β* and NO synthase activity. *Vision Research*.

[114] Kowluru RA, Odenbach S (2004). Role of interleukin-1*β* in the pathogenesis of diabetic retinopathy. *British Journal of Ophthalmology*.

[115] Vassilakopoulos T, Karatza M-H, Katsaounou P, Kollintza A, Zakynthinos S, Roussos C (2003). Antioxidants attenuate the plasma cytokine response to exercise in humans. *Journal of Applied Physiology*.

[116] Kowluru RA, Odenbach S (2004). Role of interleukin-1*β* in the development of retinopathy in rats: effect of antioxidants. *Investigative Ophthalmology & Visual Science*.

[117] Wilkinson-Berka JL (2004). Vasoactive factors and diabetic retinopathy: vascular endothelial growth factor, cycoloxygenase-2 and nitric oxide. *Current Pharmaceutical Design*.

[118] Packer L, Witt EH, Tritschler HJ (1995). Alpha-lipoic acid as a biological antioxidant. *Free Radical Biology and Medicine*.

[119] Lin J, Bierhaus A, Bugert P (2006). Effect of R-(+)-*α*-lipoic acid on experimental diabetic retinopathy. *Diabetologia*.

[120] Obrosova IG, Fathallah L, Liu E, Nourooz-Zadeh J (2003). Early oxidative stress in the diabetic kidney: effect of DL-*α*-lipoic acid. *Free Radical Biology and Medicine*.

[121] Hammes H-P, Du X, Edelstein D (2003). Benfotiamine blocks three major pathways of hyperglycemic damage and prevents experimental diabetic retinopathy. *Nature Medicine*.

[122] Sabu MC, Smitha K, Kuttan R (2002). Anti-diabetic activity of green tea polyphenols and their role in reducing oxidative stress in experimental diabetes. *Journal of Ethnopharmacology*.

[123] Mustata GT, Rosca M, Biemel KM (2005). Paradoxical effects of green tea (Camellia sinensis) and antioxidant vitamins in diabetic rats: improved retinopathy and renal mitochondrial defects but deterioration of collagen matrix glycoxidation and cross-linking. *Diabetes*.

[124] Ansari NH, Zhang W, Fulep E, Mansour A (1998). Prevention of pericyte loss by Trolox in diabetic rat retina. *Journal of Toxicology and Environmental Health—Part A*.

[125] Hammes HP, Bartmann A, Engel L, Wülfroth P (1997). Antioxidant treatment of experimental diabetic retinopathy in rats with nicanartine. *Diabetologia*.

[126] Moustafa SA (2004). Zinc might protect oxidative changes in the retina and pancreas at the early stage of diabetic rats. *Toxicology and Applied Pharmacology*.

[127] McCarty MF (2005). The putative therapeutic value of high-dose selenium in proliferative retinopathies may reflect down-regulation of VEGF production by the hypoxic retina. *Medical Hypotheses*.

[128] Kowluru RA, Engerman RL, Kern TS (1999). Abnormalities of retinal metabolism in diabetes or experimental galactosemia. VI. Comparison of retinal and cerebral cortex metabolism, and effects of antioxidant therapy. *Free Radical Biology and Medicine*.

[129] Garay RP, Hannaert P, Chiavaroli C (2005). Calcium dobesilate in the treatment of diabetic retinopathy. *Treatments in Endocrinology*.

[130] Spadea L, Balestrazzi E (2001). Treatment of vascular retinopathies with *Pycnogenol*. *Phytotherapy Research*.

[131] Bursell S-E, Clermont AC, Aiello LP (1999). High-dose vitamin E supplementation normalizes retinal blood flow and creatinine clearance in patients with type 1 diabetes. *Diabetes Care*.

[132] Millen AE, Gruber M, Klein R, Klein BEK, Palta M, Mares JA (2003). Relations of serum ascorbic acid and *α*-tocopherol to diabetic retinopathy in the Third National Health and Nutrition Examination Survey. *American Journal of Epidemiology*.

[133] Millen AE, Klein R, Folsom AR, Stevens J, Palta M, Mares JA (2004). Relation between intake of vitamins C and E and risk of diabetic retinopathy in the Atherosclerosis Risk in Communities Study. *American Journal of Clinical Nutrition*.

[134] Mayer-Davis EJ, Bell RA, Reboussin BA, Rushing J, Marshall JA, Hamman RF (1998). Antioxidant nutrient intake and diabetic retinopathy: the San Luis Valley diabetes study. *Ophthalmology*.

